# Inter- and intra-breed genome-wide copy number diversity in a large cohort of European equine breeds

**DOI:** 10.1186/s12864-019-6141-z

**Published:** 2019-10-22

**Authors:** Marina Solé, Michela Ablondi, Amrei Binzer-Panchal, Brandon D. Velie, Nina Hollfelder, Nadine Buys, Bart J. Ducro, Liesbeth François, Steven Janssens, Anouk Schurink, Åsa Viklund, Susanne Eriksson, Anders Isaksson, Hanna Kultima, Sofia Mikko, Gabriella Lindgren

**Affiliations:** 1 Department of Animal Breeding & Genetics, Swedish University of Agricultural Sciences, Uppsala, Sweden; 2 Department of Veterinary Science, Università di Parma, Parma, Italy; 3 Department of Medical Sciences, Array and Analysis Facility, Uppsala University, Uppsala, Sweden; 4 Faculty of Life and Environmental Science, University of Sydney, Sydney, NSW Australia; 5 Livestock Genetics, Department of Biosystems, KU Leuven, 3001 Leuven, Belgium; 6 Animal Breeding and Genomics, Wageningen University & Research, P.O. Box 338, 6700 AH Wageningen, the Netherlands; 7 Centre for Genetic Resources, the Netherlands (CGN), Wageningen University & Research, P.O. Box 338, 6700 AH Wageningen, the Netherlands

**Keywords:** Copy number variation, Horse, Structural variation, SNP genotyping array

## Abstract

**Background:**

Copy Number Variation (CNV) is a common form of genetic variation underlying animal evolution and phenotypic diversity across a wide range of species. In the mammalian genome, high frequency of CNV differentiation between breeds may be candidates for population-specific selection. However, CNV differentiation, selection and its population genetics have been poorly explored in horses.

**Results:**

We investigated the patterns, population variation and gene annotation of CNV using the Axiom® Equine Genotyping Array (670,796 SNPs) from a large cohort of individuals (*N* = 1755) belonging to eight European horse breeds, varying from draught horses to several warmblood populations. After quality control, 152,640 SNP CNVs (individual markers), 18,800 segment CNVs (consecutive SNP CNVs of same gain/loss state or both) and 939 CNV regions (CNVRs; overlapping segment CNVs by at least 1 bp) compared to the average signal of the reference (Belgian draught horse) were identified. Our analyses showed that *Equus caballus* chromosome 12 (ECA12) was the most enriched in segment CNV gains and losses (~ 3% average proportion of the genome covered), but the highest number of segment CNVs were detected on ECA1 and ECA20 (regardless of size). The Friesian horses showed private SNP CNV gains (> 20% of the samples) on ECA1 and Exmoor ponies displayed private SNP CNV losses on ECA25 (> 20% of the samples). The Warmblood cluster showed private SNP CNV gains located in ECA9 and Draught cluster showed private SNP CNV losses located in ECA7. The length of the CNVRs ranged from 1 kb to 21.3 Mb. A total of 10,612 genes were annotated within the CNVRs. The PANTHER annotation of these genes showed significantly under- and overrepresented gene ontology biological terms related to cellular processes and immunity (Bonferroni *P*-value < 0.05). We identified 80 CNVRs overlapping with known QTL for fertility, coat colour, conformation and temperament. We also report 67 novel CNVRs.

**Conclusions:**

This work revealed that CNV patterns, in the genome of some European horse breeds, occurred in specific genomic regions. The results provide support to the hypothesis that high frequency private CNVs residing in genes may potentially be responsible for the diverse phenotypes seen between horse breeds.

## Background

With the development of genome-wide genotyping arrays, genetic diversity and the variation of complex phenotypes are often assessed via single nucleotide polymorphism (SNP). However, variation in SNPs only explains a fraction of the genetic component of phenotypic variance. Recently, increased attention has been drawn to copy number variations (CNVs), as they are widely considered to impact phenotypes. CNVs are typically defined as variation due to deletions, insertions, and duplication events (1 kilobase-pairs (kb) to several megabase-pairs (Mb)) when comparing the genomic sequence of individuals with a reference genome [1].

The total number of CNVs could explain much of the heritability unaccounted for by SNPs as CNVs have been linked to genetic variation of complex traits influencing phenotypic diversity and disorders across a wide range of species [2–11]. In horses, it has been shown that CNVs can cause phenotypic variation and disorders. A 4.6-kb intronic duplication in *STX17* on ECA25 has been linked to hair greying and melanoma [12], and large deletions at the *SHOX* locus in a pseudoautosomal region are associated with Skeletal Atavism in Shetland Ponies [13]. Furthermore, studies in dogs have been shown that between 3 and 24% of unique CNVs potentially contribute to phenotypic diversity [9]. For instance, the breed characteristic dorsal hair in Rhodesian and Thai Ridgebacks has been linked to a duplication of a set of *FGF* genes, which also predispose to dermoid sinus disorder [11].

There are also some mammalian gene super-families commonly known to underpin evolutionary changes driven by CNVs, where genes associated to CNV gains may be a likely instrument of adaptation [14]. Consequently, most of the variation explained by CNVs in the mammalians’ genomes is known to occur in regions that regulate important biological processes such as sensory perception, signal transduction, immunity and pathogen defence or metabolism pathways [2, 5, 7–9, 14]. Therefore, the analysis of CNVs in domestic and livestock species has become increasingly important for the evaluation of genetic diversity, phenotypic variation and complex phenotypes.

The current publicly available database of genetic variants in the equine genome contains 25,756,212 SNP and 3,663,455 insertion/deletions polymorphisms “INDELs”, accessed from the Genome Variation Map (http://bigd.big.ac.cn/gvm/home). Until now, CNVs identified in nearly 45 different horse breeds occupy about 1–3% of their genome and there are more CNVs residing in genes (~ 80%) than in intergenic regions (~ 20%) [5, 7, 15–19]. In horses, the average range for CNV size remains between 1 kb to 4.84 Mb with CNV losses generally dominating over gains in comparison with a reference Thoroughbred genome [7, 15–17]. The majority of the actual number of identified specific CNVs in horses has been reported using a limited number of individuals [7, 17, 20]. Although significant associations with specific traits such as body size or recurrent laryngeal neuropathy have been detected using large sample datasets [18, 19].

Previous studies in other mammals have hypothesized that CNVs with high frequency differences among breeds are involved in population-specific selection and adaptation to the environment [21–23], and also that CNVs residing in genes contribute more to population differentiation and divergence [24]. However, CNV differentiation, selection and its population genetics have been poorly explored in horses. As such, in the present study we aimed to 1) investigate the distribution pattern of CNVs, 2) detect breed specific CNVs and 3) identify biological pathways affected by CNVs in the horse genome using a large cohort of individuals and high density (HD)-SNP genotyping array data across eight European equine breed populations (Ardenner, Belgian draught, German draught, Exmoor, Vlaams paard, Friesian horses, Belgian Warmblood and Swedish Warmblood).

## Results

### Copy number variations in various European horse populations

Genome-wide CNV analysis was conducted on 1755 horses from 8 different breeds (Table 1) using the Affymetrix Axiom™ Equine HD-SNP genotyping array (MNEC670K) [25]. CNVs were analysed on autosomes by comparing the ratio of signal intensities of each sample with the average signal of the reference (Belgian draught horse; extensive sample size (*N* = 301) and the lowest variation in signal intensity among the studied breeds). After quality control, we identified 152,640 SNP CNVs (signal gains or losses specific to each individual and state), 18,800 segment CNVs (consecutive SNP CNVs per individual of same gain/loss state or both), and 939 CNV regions (CNVRs; overlapping segment CNVs by at least 1 bp in the whole population per state: signal gain, loss or both).

**Table 1:** Descriptive statistics for Copy Number Variations (segment CNVs)

Breeds	N of individuals	% total change	sd total change	% gain	sd gain	% loss	sd loss
*Draught*							
Ardenner	24	0.2	0.05	0.1	0.05	0.1	0.04
Belgian draught^a^	301	0.2	0.23	0.1	0.07	0.1	0.22
German draught	22	0.2	0.09	0.1	0.08	0.1	0.04
Exmoor ponies	256	0.2	0.43	0.2	0.41	0.1	0.10
Vlaams paard	22	0.2	0.08	0.2	0.07	0.1	0.03
*Warmblood*							
Belgian Warmblood I	234	0.3	0.11	0.2	0.12	0.1	0.04
Belgian Warmblood II	247	0.4	0.92	0.3	0.84	0.1	0.09
Swedish Warmblood	383	0.3	0.21	0.2	0.19	0.1	0.08
*Friesian*							
Friesian horses	266	0.9	1.10	0.7	1.10	0.1	0.12

Average and standard deviation (sd) of the proportion of the total genome (without sex chromosomes) with a percent total change of segment CNVs, percentage gains of segment CNVs, and percent losses of segment CNVs, compared to the average signal of the reference^a^

**Fig. 1:**
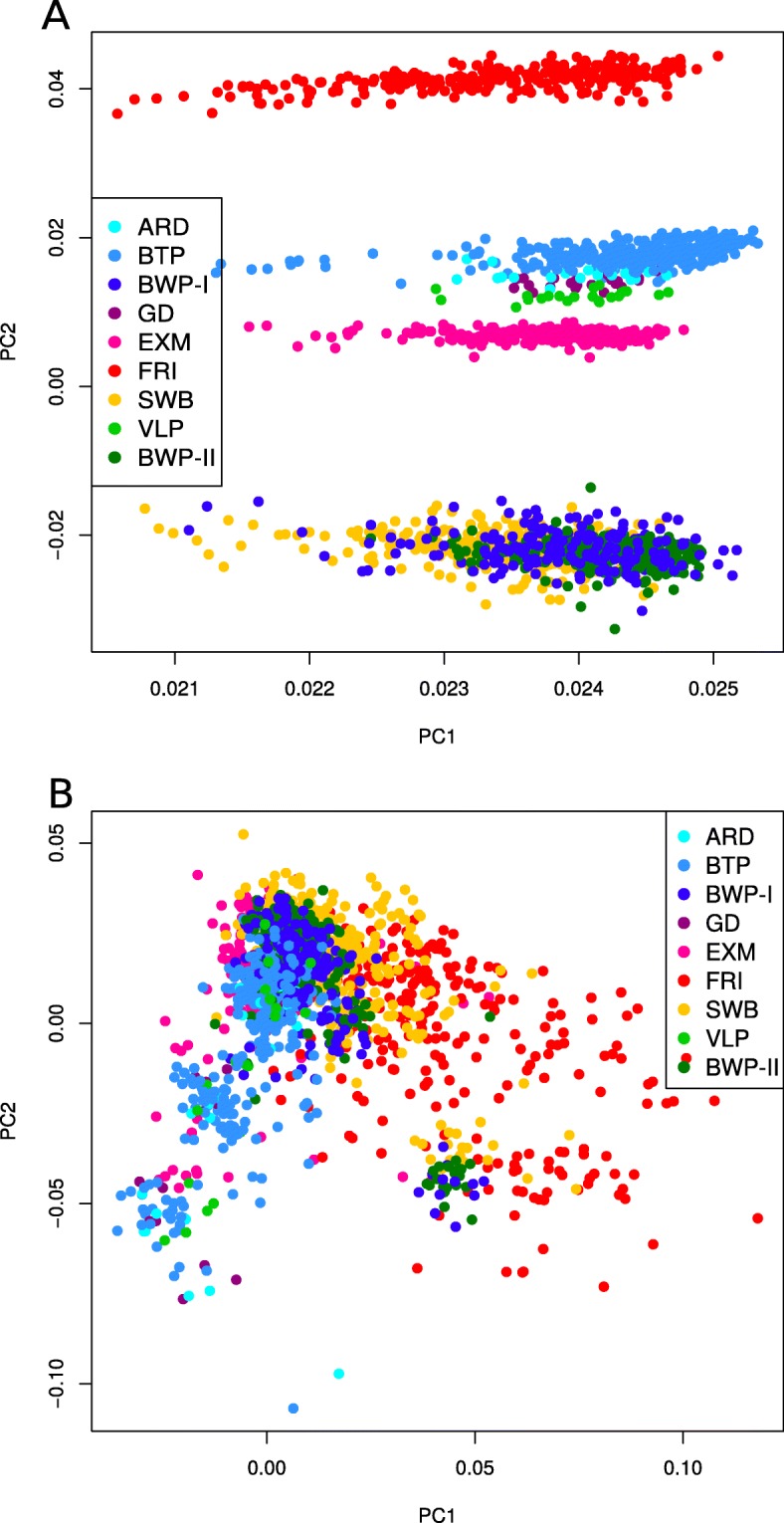
Principal component analysis across the eight European horse breeds. **a** (left) SNP B-allele frequency ratios of the samples. **b** (right) CNV of the segments detected, compared to the average signal of the reference. ARD = Ardenner; BTP = Belgian draught horse (*reference); BWP-I and BWP-II = Belgian Warmblood I and II; GD = German draught; EXM = Exmoor ponies; FRI = Friesian horses; SWB = Swedish Warmblood; VLP = Vlaams paard

To shed light on the diversity between breeds and their CNVs distribution, we used principal component analysis (PCA). First, the plot for the SNP B-allele frequency ratios (normalized measure of the allelic intensity ratio of the two alleles) showed a clear separation of the individuals in three main breed groups on PC2, with the Friesian horses uniquely distinguished from the other horse breed populations, that clustered in two main subpopulations; *i*) Warmblood horses, and *ii*) Draught horses including Exmoor ponies (Fig. 1a).

Interestingly, the first principal component represented variation between individuals, across breeds (PC1 explained 61.9% of the total variance), and the second principal component represented variation between breeds (PC2 explained 3.1% of the total variance). In contrast, PCAs generated from segment CNVs showed less variation across breeds (Fig. 1b). However, Friesian horses were the most differentiated with 0.9% of total segment CNV changes in their genome when compared to the reference (Table 1).

**Fig. 2:**
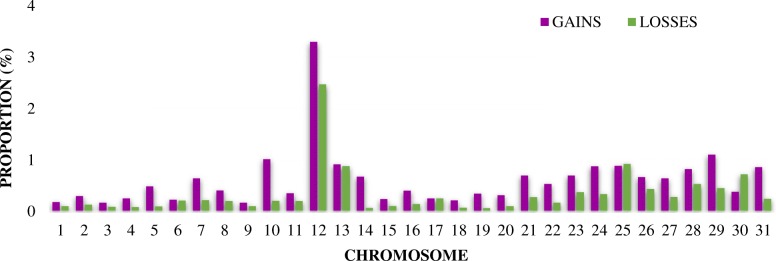
Chromosomal distribution of the detected segment CNVs. Length of colored bars corresponds to the average proportion of the genome covered in percentage. Segment CNVs gains (purple), segment CNVs losses (green), compared to the average signal of the reference

**Fig. 3:**
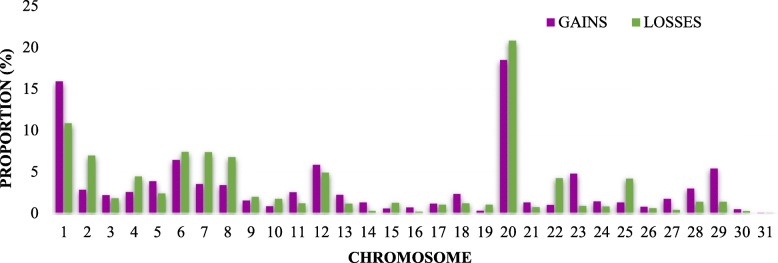
Chromosomal distribution of the detected segment CNVs, regardless of the chromosomal genome size. Length of colored bars corresponds to the percentage of number of segment CNVs at a particular chromosome. Segment CNVs gains (purple), segment CNVs losses (green), compared to the average signal of the reference

The distribution of segment CNVs showed several overlapping regions in the genome of the studied breeds. The largest percentage of the genome covered by segment CNV gains and losses (~ 2.5–3.0% average percentage of the genome covered; Fig. 2) was detected in ECA12, but the highest percentages of number of segment CNVs (above 10%) were detected on ECA1 and ECA20 (regardless of the chromosomal genome size; Fig. 3).

### Private CNV comparisons

**Fig. 4:**
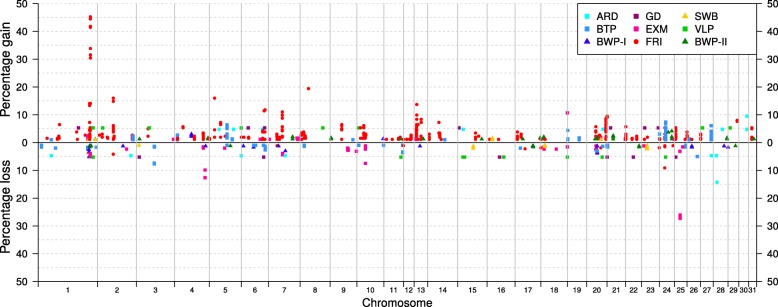
Chromosomal percentage distribution of private SNP CNV gains and losses. SNP CNV detected in at least 1% of the samples within breed, compared to the average signal of the reference. ARD = Ardenner; BTP = Belgian draught horse (*reference); BWP-I and BWP-II = Belgian Warmblood I and II; GD = German draught; EXM = Exmoor ponies; FRI = Friesian horses; SWB = Swedish Warmblood; VLP = Vlaams paard

The number of locations with unique private CNV gains and losses relative to the reference breed varied between the populations. In total, the number of genome positions that displayed SNP CNV gains ranged between 4629 (Ardenner) and 56,033 (Friesian horses) (Table 2). The average number of SNP CNV gains per individual ranged between 25.14 (Belgian draught horse) and 332.64 (Ardenner). The number of genome positions observed with SNP CNV losses ranged between 3715 (Vlaams paard) and 7142 (Friesian horses) (Table 2), whereas the average number of SNP CNV losses per individual ranged between 13.22 (Swedish Warmblood) and 168.86 (Vlaams paard). In this sense, percentage of unique private SNP CNV gains in relation to the total number of gains within breed ranged between 0.1% (Belgian warmblood I) and 10.5% (Friesian horses), and percentage of unique private SNP CNV losses in relation to the total number of losses ranged between 0.4% (Ardenner) and 14.0% (Exmoor ponies). The within population percentage, and positions of the unique private SNP CNVs are plotted in Fig. 4. The Friesian horses showed the largest percentage of unique private SNP CNV gains (> 20% of the samples) on ECA1 and Exmoor ponies displayed the largest percentage of unique private SNP CNV losses on ECA25 (> 20% of the samples).

**Table 2:** Breed total number of genome positions that display SNP copy number gains and losses

Breeds	SNP CNV		% Unique private CNV^b^	
	gains	losses	gains	losses
*Draught*				
Ardenner	4629 (192.88)	4324 (180.17)	3.1	0.4
Belgian draught horse^a^	7568 (25.14)	5894 (19.58)	2.8	2.5
German draught	5777 (262.59)	3894 (177.00)	2.5	0.6
Exmoor ponies	18,150 (70.90)	4236 (16.55)	6.5	14.0
Vlaams paard	7318 (332.64)	3715 (168.86)	2.9	1.2
*Warmblood*				
Belgian Warmblood I	9837 (42.04)	5760 (24.62)	0.1	1.5
Belgian Warmblood II	10,852 (43.94)	5473 (22.16)	2.4	1.6
Swedish Warmblood	13,994 (36.54)	5064 (13.22)	0.9	1.1
*Friesian*				
Friesian horses	56,033 (210.65)	7142 (26.85)	10.5	1.4

SNP CNVs compared to the average signal of the reference^a^. The average number of SNP CNVs / individual is given within brackets. The percentage of breed specific (unique private) SNP CNV gains and losses in the genome are also shown. ^b^The sex chromosomes are not included in the total genome size. Every genome position showed a particular SNP CNV in at least 1% of the samples within breed

### Breed cluster CNV comparisons

To better understand patterns of CNVs between breeds of common ancestral origin, we grouped the breeds in three breed clusters according to the previous PCA analysis (Fig. 1a; Draught including Exmoor ponies, Warmblood and Friesian horses [26]). The total number of genomic locations presenting SNP CNVs (signal gains and losses) and the percentage of unique private SNP CNV gains or losses corresponding to the breed group clusters are shown in Table 3. The Friesian horses showed the largest average number of SNP CNV gains and losses per individual (210.65 and 26.85, respectively). The percentage of unique private SNP CNV gains in relation to the total number of gains within breed group was higher in the Friesian horses (10.5%), and the percentage of unique private SNP CNV losses in relation to the total number of losses was similar in the Draughts and Warmbloods (18.7 and 19.2%, respectively).

**Table 3:** Breed group total number of genome positions that display SNP copy number gains and losses

Breeds	N of individuals	SNP CNV		% Unique private CNV^a^	
		gains	losses	gains	losses
Draught	624	7527 (12.06)	6041 (9.68)	1.99	18.66
Warmblood	864	13,133 (15.20)	5591 (6.47)	8.62	19.19
Friesian	266	56,033 (210.65)	7142 (26.85)	10.47	1.43

SNP CNVs compared to the average signal of the reference (Belgian draught horse). The average number of SNP CNVs / group cluster is given within brackets. The percentage of group specific (unique private) SNP CNV gains and losses in the genome are also shown. ^a^The sex chromosomes are not included in the total genome size. Every genome position showed a particular SNP CNV in at least 1% of the samples within breed group

**Fig. 5:**
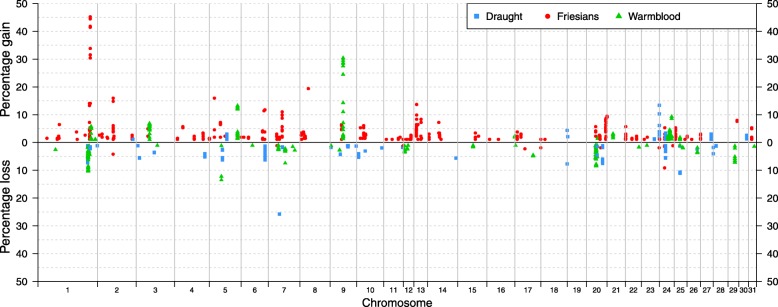
Chromosomal percentage distribution of breed cluster-specific SNP CNV gains and losses. SNP CNV detected in at least 1% of the samples within breed clusters, compared to the average signal of the reference

The chromosomal percentage distribution of unique private SNP CNV gains and losses across the entire genome of the three breed clusters is shown in Fig. 5. Particularly, the Friesian horses showed a high percentage of unique private SNP CNVs gains (> 20% of the samples) at the same location shown in previous plot (ECA1, Fig. 3). The Warmbloods showed genomic regions with high percentage (> 20% of the samples) of unique private SNP CNV gains in ECA9 (Fig. 5) compared to the reference. The Draughts also showed regions with high percentage (> 20% of the samples) of unique private SNP CNV losses in ECA7 (Fig. 5) compared to the reference.

### CNV regions, gene annotation and PANTHER classification

In the present study, we identified 18,800 segment CNVs representing 13,178 segment CNV gains (average length of 414.78 kb), 5298 segment CNV losses (average length of 196.95 kb) and 324 segment CNV gains/losses (average length of 241.84 kb), compared to the reference. Overlapping segment CNVs by at least 1 bp were merged into 939 CNVRs (ranging from 1 kb to 21.3 Mb length; Additional file 1: Table S1). The majority of CNVRs contained genes (79.3%). Specifically, 10,612 genes were annotated within the CNVRs (Additional file 2). The PANTHER analysis showed significantly underrepresented gene ontology (GO) terms involved in single-stranded RNA binding, transposition, cellular response to lipopolysaccharide and ribonucleoprotein complex functions (Bonferroni *P*-value < 0.05; Additional file 3: Table S2). Overrepresented GO terms involved in G-protein coupled receptor activity, immune response, immunoglobulin and membrane-bound signalling molecule functions (Bonferroni P-value < 0.05; Additional file 3: Table S2). Additionally, we identified 80 CNVRs (ranging from 129 kb to 9.04 Mb length) which overlapped with known QTLs for conformation, coat colour, fertility, hair and temperament traits (Additional file 1: Table S1).

### Novel CNVR and validation by RT-qPCR

The majority of the identified CNVRs overlapped with at least 1 bp with the previously identified and published equine CNVRs, while 67 out of 939 CNVRs were novel (7.1%, (see Methods and Additional file 1: Table S1). To further verify the accuracy of CNVR prediction, RT-qPCR was used to validate four genomic regions containing common segment CNVs of different sizes detected in this study (from 12 kb to 2 Mb length). The CNVR located within *Olfr4F21* gene on ECA1 and *OR8S1* gene on ECA6 were already detected in previous studies [15, 20], whereas the remaining two were novel CNVRs. The CNVRs represented a different state of the segment CNVs (signal gain and loss in relation to the average signal of the reference; Additional file 4: Table S3). From the 80 sample-locus combinations tested, all four CNVRs were confirmed by RT-qPCR, as different copy number states were found per each CNVR.

Genotyping concordance rate between the Affymetrix Axiom array data and the RT-qPCR validation differed slightly between regions (Additional file 4: Table S4). In the case of the CNVR within the *Olfr4F21* gene, the concordance rate was equal to 90% with one duplication in one horse not confirmed by RT-qPCR. Both the CNVRs located within *OR10G2* and *OR08S1* genes had a concordance rate of 80%. For the CNVR located within the *OR08S1* gene, two individuals showed homozygous one copy deletion by RT-qPCR which were not detected by the HD-SNP genotyping array. One deletion was not confirmed by RT-qPCR in the case of *OR10G2* gene and one duplication was found by RT-qPCR which was not previously detected from the SNP array. In the case of the CNVR located in the *SV2C* gene, two samples showed homozygous one copy deletion in the RT-qPCR which were not detected from the HD-SNP genotyping array.

## Discussion

### Diversity of CNVs between different European horse breeds

Genetic polymorphisms play an important role in the phenotypic diversification and speciation in equids [27, 28]. Although diverse genetic variants underlying phenotypic variation have been successfully mapped (e.g. [12, 13, 29, 30]), a large proportion of the horse genome still remains poorly understood. Using HD-SNP array data from a large cohort of individuals across groups of phenotypically and ancestrally divergent horses, we showed that CNV distribution across different breeds presented many commonalities (genomic location, gain or loss), but that some unique private CNVs were observed in particular genomic regions. Moreover, both validation rate of CNVRs and overall genotyping concordance rate of 82.5% proved the Axiom Analysis as a consistent method for CNV calling.

Principal component analysis showed remarkable variation among populations, and was in accordance with known breed divergence and history [26, 31]. Comparable findings were also pointed out in other domestic and livestock species [23, 32, 33]. Similar to the current study, previous studies have indicated clear distinctions in CNV frequency between breed groups or populations, possibly reflecting breed patterns of phenotypic diversity and the population history of different breeds, such as a change in past effective population size, gene flow, or selection [9, 21, 23]. Our observed differences also support the hypothesis that genetic variation from CNVs may contribute to breed phenotypic diversity, but it may also result from the differential demographic history and effective population sizes between breeds [9, 23].

With only minor exceptions, the CNV distribution, showed small differences in SNP and segment CNVs between breeds. The average percentage of total coverage by the segment CNVs identified across the genome of the investigated breeds was small in relation to the reference breed (< 0.5%). In general, low CNV diversity is expected [7] as the segment CNVs identified in the present study covered an even smaller proportion of the genome in comparison with those previously reported in the same type of horse breeds [5, 7, 16, 17]. Consequently, the differences observed may be attributable to the different genetic background of the individuals, sample size and methodologies applied for CNV discovery. As observed in other studies with the same type of horse breeds, the largest number of shared CNVRs were found in ECA12 (e.g. ECA12:9,158,392-17,707,943 [5, 7, 16, 18, 20]). ECA12 displays the particular feature of being enriched with clusters of olfactory receptor genes, which is also observed in other mammalian genomes and it has been hypothesized to influence flight response and temperament diversity in horses [7]. Similarly, we found overlapping of CNVRs with previously identified T-cell receptor and MHC class genes, that exhibited high levels of diversity in one or more similar type of horse breeds (e.g. on ECA1:154,857,175-156,876,500; ECA1:158,843,180-160,751,024; and ECA20:28,731,700-35,604,382) [5, 7, 16, 18]. Interestingly, ECA1 overlapped with established QTLs for conformation in British ponies and reproductive traits in German Warmbloods (e.g. on ECA1:53,917,112-54,139,087 and ECA1:93,506,091-95,186,154 [34, 35]), and CNVRs in ECA20 overlapped with QTLs for back conformation in American Saddlebred horses (e.g. on ECA20:41,994,712-43,192,412 and ECA20:43,299,741-43,568,400 [36]). The largest amount of shared CNVRs within all horse breeds studied overlapped with QTLs for white markings detected in the light draught Franches-Montagnes horses (ECA1:154,857,175-156,876,500 [37]) and withers at height detected in British ponies (ECA7:43,0483,386-53,094,544; ECA8:0–6,769,072 and ECA12:9,158,392-17,707,943 [34]). Although no candidate genes have been previously reported in these regions, our findings suggest that the functionality of CNV-enriched genes in horses fall into sensory perception, immunity, reproduction and exterior traits.

Our results also support the hypothesis that high frequency private SNP CNVs in particular (e.g. on ECA25 in Exmoor ponies) may be responsible for population-specific selection and adaptation [21, 22]. This provides further evidence to presume that CNVs in these regions may represent a substantial source of genetic variation for diverse phenotypes and biological processes, although further analysis is required to confirm phenotypic changes. Additionally, a greater amount of segment CNV gains compared to losses was observed, potentially reflecting the large number of losses in the reference native Belgian draught horse. However, this may also reflect the fact that duplications of coding sequences potentially enhance the organisms’ genetic diversity, phenotypic variation and adaptation potential [38, 39].

### Diversity of CNVs in breed clusters

The large cohort of individuals analysed in this study (~ 195 horse on average per breed), in comparison with other studies where breeds are represented with one or two horses, is likely to provide a more accurate overview of single private CNVs. The PCA analysis of the SNP and segment CNV distribution according to the three breed clusters confirmed similar frequency levels within three breed clusters. This should not be surprising given that the largest number of shared CNVs was previously reported between closely related breeds such as Warmbloods [18].

Our results also showed that the proportion of breed cluster-specific SNP CNVs differed between groups. For instance, the Draught and Warmblood clusters displayed a relatively high proportion of unique private SNP CNVs (up to 30–50%). However, a smaller proportion of SNP CNVs may be attributable to breed-specific characteristics (less than 14%). Such differences in unique CNVs in ancestrally divergent *Equidae* members have also been previously reported. As an example, Doan et al. [7] detected higher proportions of certain specific CNVs in donkeys (35%) and miniature (24%) horses, over the total breed-specific CNVs across 17 different *Equidae* species, which has been attributed to larger divergences relative to the Thoroughbreds.

Interestingly, we also identified breed group-specific SNP CNVs located in particular genomic regions which may be attributable to breed-group features. For instance, specific SNP CNVs gains were detected in ECA1 (163,032,489-163,181,822) in more than 20% of the individuals belonging to the Friesian horses’ cluster, and more than 20% of the individuals belonging to the Draught cluster showed specific SNP CNV losses in ECA7 (34,601,429-34,608,700). However, all these private SNP CNVs identified reside in intergenic regions or merge into CNVR which partially overlap with previously identified regions in the Quarter horse [17]. Notwithstanding, the identified SNP CNVs gains in ECA9 (40,822,784-40,867,675) in more than 20% of the individuals belonging to the Warmblood cluster reside in a region which contain two genes, RAD54 homolog B *(RAD54B)* and Reactive intermediate imine deaminase A *(RIDA)*. The latest is regarded as a potential candidate gene for athletic performance since it is involved in metabolic processes and has been related to blood protein levels in humans [40]. CNV polymorphisms in these region may represent a substantial source of genetic variation of high value for genetic association analyses in the future.

### PANTHER analysis of genes underlying the CNVR and novel CNVR discovery

The evolutionary process of species formation (speciation) is complex and influenced by fast evolving changes in specific regions in the genome (i.e. CNVRs driving novel gene functions), which may affect regulatory key biological mechanisms and play a fundamental role in gradual adaptation to different environments [1, 22]. In the last 10 years, substantial progress has been made in relation to the functional impact of structural variants in several species with focus on population diversity. Enrichment of CNVRs in genes related to immune response, brain development, metabolic processes, sensory perception of smell or chemical stimuli have been reported in global populations of humans as well as pigs, dogs, cattle and horses [2, 4, 8, 18, 23, 41]. In this sense, our results also indicate that CNVRs are located in specific genomic regions and are involved in important biological processes in mammals such as immunity. As horse populations have gone through strong and diverse selection since horse domestication [28], these findings could be expected and may indicate favourable selection of structural variants associated with specific traits (e.g. insect bite hypersensitivity (IBH) in the Friesian horses [20]). However, the association of CNVs with certain private traits in horses needs further exploration.

## Conclusions

Our PCA analysis of SNP and segment CNVs showed that horse populations tend to group according to breed ancestral origins. Comparing breed and breed group clusters, we identified potentially unique private genomic regions displaying SNP CNVs. We found small percentages (less than 14%) of unique private intra-breed structural variants that may contribute to the equine breed diversity. Specifically, ECA9 displayed a high frequency of specific SNP CNVs gains in the Warmblood cluster in a region containing *RIDA* gene, involved in metabolic processes. Genes located within CNVRs demonstrated under- and overrepresentation of gene ontology biological terms related to cellular processes and immunity, suggesting the potential role of structural variants in driving phenotypic diversity and disease resistance in European horse populations. We identified 80 CNVRs overlapping with established fertility, coat colour, conformation and temperament trait QTLs. We also report 67 novel CNVRs, which contribute to the catalogue of known CNVs in the horse genome. Future research is needed in order to confirm if the observed CNVs across breeds are also linked to phenotypical differences.

## Methods

### Horses, breeds

The studied cohort comprised 1755 horses representing eight breeds (Table 1). The Friesian horse samples belonged to a half-sib case-control set-up to study IBH [20]. The Exmoor ponies had previously been genotyped for a case-control genome scan to detect genetic risk factors for IBH [42]. The Swedish Warmblood horses were selected randomly from four different performance groups representative of the SWB population in general. The rest of the horse breeds belonged to case-control set-ups for different diseases under the EU HORSEGENE project. Belgian Warmbloods were treated initially as 2 cohorts (BWP-I and BWP-II) as they differed in average birth year by 9 years (approx. 1 generation). Based on clustering results, all Warmbloods were merged together.

### DNA isolation

Genomic DNA from the Friesian horses and Exmoor ponies was isolated as described in Schurink et al. [20] and Velie et al. [42]. DNA for Swedish Warmbloods was prepared from 20 hair roots, cut into 5% Chelex 100 Resin (Bio-Rad Laboratories, Hercules, CA, US), and 1.4 mg/ml Proteinase K (Merck KgaA, Darmstadt, Germany) in a total volume of 200 μl. The samples were incubated at 56 °C, 1500 rpm for 2 h, followed by heat inactivation of Proteinase K at 96 °C for 10 min. DNA concentration was normalized, and the DNA was re-suspended in lowTE (1 mM Tris, 0.1 mM EDTA) at a concentration of 10 ng/μl. The QIAamp DNA Blood Midi Kit was used to isolate DNA from whole blood of the Belgian Warmblood, Belgian Draught, German Draught, Vlaams paard and Ardenner horses. Lower limits for genotyping where a concentration of at least 20 ng/μl, a total amount of 300 ng DNA per sample and an OD ratio 260/280 between 1.8–2.0 was used.

### Microarray analysis

Genotyping was performed according to the standard protocol Axiom™ 2.0 Assay Manual Workflow User Guide (P/N 702990, ThermoFisher Scientific, Life Technologies, Carlsbad, CA 92008 USA). Briefly, 100 ng of total genomic DNA was denatured and then amplified. The amplified DNA was fragmented, precipitated and then centrifuged. The pellets were dried and resuspended in a buffer and added to a hybridization master mix, followed by hybridization to the microarray plate (Axiom™ Equine Genotyping Array), in the GeneTitan™ Multi-Channel Instrument for 23.5 h. Moreover, the array plate was labelled with biotin and FAM, washed and further stained with streptavidin and αFAM antibody. Finally, scanning was performed in the Gene Titan™ Multi-Channel Instrument.

### CNV data analysis and QC

Copy number calling was performed in the Axiom™ Analysis Suite 1.1 according to the Best Practices Analysis Workflow described in Axiom™ Genotyping Solution Data Analysis Guide (P/N 702961, ThermoFisher Scientific, Life Technologies, Carlsbad, CA 92008 USA) [43]. The threshold for the Dish QC quality metric was set to 0.82 according to the manufacturer’s instructions. Following standard protocol, only the samples passing Call Rate > 96% were retained. The Axiom® CNV summary software tool was used to generate input files for CNV prediction analysis, which were further processed using Nexus Copy Number™ 9.0 (BioDiscovery, Hawthorne CA 90250, U.S.A.).

### Determination of population structure and group-specific CNV clusters

Principal component analysis (PCA) of the SNP B-allelic frequency ratios of the samples and signal intensity of the copy number changes of the sample segments was done using the prcomp function in R v13.1 [44].

For the detection of breed specific SNP CNVs, we defined any SNP CNV identical between two or more horses across breeds as a shared SNP CNV. Similarly, for the detection of breed group-specific SNP CNVs, the eight breeds were grouped into three breed clusters according to the PCA analysis: Draught (Ardenner, Belgian draught, German draught, Exmoor ponies, Vlaams paard), Warmbloods (Belgian Warmblood and Swedish Warmblood), and Friesian horses. The Belgian draught horses showed the least variation in SNP CNVs and had a large sample size among the breeds studied. Therefore, the mean signal intensity of these individuals was used as a reference to discriminate between CNV gain (increased signal in relation to the average) and CNV loss (decreased signal in relation to the average).

The analysis of unique private SNP CNVs within breed groups was done using in house scripts, considering any SNP CNVs detected only in a single breed group (not shared with other breed groups) as a “unique private” breed group-specific SNP CNV.

### Determination of CNVR, gene annotation and PANTHER analysis

First, those segment CNVs that overlapped by at least 1 bp were summarized as a single CNVR using the BEDTools software (−merge Bed command) [45]. Second, we determined the overlap (when at least 1 bp was in common) between CNVRs identified in our study with the CNVRs already available online (DGVa, https://www.ebi.ac.uk/dgva) or published in recent scientific literature [20] on different population sizes and methods using BEDTools software (−intersect Bed command). This approach allowed us to identify CNVRs that had not been discovered so far (i.e. novel CNVRs) and CNVRs that are common between a wide range of equine breeds worldwide. The overlap between the CNVRs identified in the whole cohort of horses studied and genes annotated within the CNVRs in the horse genome (based on the EquCab2.0 sequence assembly), was determined using Variant Effect Predictor (VEP [46];) from the bioinformatics database Ensembl (http://www.ensembl.org/). Overrepresentation test was performed using the PANTHER (Protein ANalysis THrough Evolutionary Relationships, version 11.0; http://www.pantherdb.org/) classification system for the classification of genes by their molecular function, biological process, cellular component and protein class with default Bonferroni correction and false discovery rate (FDR) [47]. The identified CNVRs were also compared to previously reported trait QTLs in the horse (downloaded from the horse QTL database [48];) using bed file comparisons in BEDTools software (−merge Bed command) [45].

### Validation by RT-qPCR

Validation of four genomic regions containing CNVRs was performed by real-time quantitative PCR (RT-qPCR) on the StepOne™ Real-Time PCR System (Applied Biosystems by Life Technologies, Darmstadt, Germany). These CNVRs were located within annotated genes. Primers and probes were designed using the Custom TaqMan® Assay Design Tool (Additional file 4: Table S3). Twenty horses were available for validation which belonged to three breeds: Exmoor ponies, Swedish Warmblood horses and Friesian horses. Ten out of the 20 horses were used in the CNVRs calling from the Axiom Analysis, thus allowing direct comparison of the results. DNA was prepared from 20 hair roots as described above (see Swedish Warmblood DNA Isolation protocol). DNA quality was determined with a Qubit® Fluorometer. For each CNVR, 4 different concentrations were used to determine assay efficiency: 50 ng/μL, 25 ng/μL, 12.5 ng/μL and 6.25 ng/μL of DNA. Reactions were assembled in a final volume of 15.00 μL, containing 1.50 μL gDNA, 7.55 μL PCR Master Mix 2X and 0.75 μL of Custom TaqMan® Assay mix 20X and 5.20 μL of nuclease free water. The RT-qPCR conditions were as follows: initial denaturation at 95 °C for 10 min (min), followed by 40 cycles of denaturation at 95 °C for 15 s and combined annealing and extension at 60 °C for 1 min. Analysis was performed per each horse in duplicate and the average value was used for further calculations. The fold changes were determined using the 2^-ΔΔCt^ method that normalises the C_t_ values (cycle threshold) of the target gene with a reference gene (ΔC_t_), and compare the ΔC_t_ to the ΔC_t_ of the reference sample [49]. We used as reference gene the Glyceraldehyde-3-phosphate dehydrogenase (*GAPDH*) gene on ECA6, according to previous CNVRs analyses in horses [18]. The reference individual was chosen among the 1755 horses and it displayed neither deletions nor duplications in the CNV prediction from the array in any of the four investigated CNVRs.

## Supplementary information

**Additional file 1: Table S1.** CNVRs identified in this study and including information regarding genome position, size, status, segment CNV counts, gene counts, number of individuals for each breed, QTL overlap and novelty.

**Additional file 2.** List of genes overlapping with the identified CNVRs.

**Additional file 3: Table S2.** PANTHER gene ontology analysis of significantly over- and underrepresented genes.

**Additional file 4: Table S3.** Genomic regions validated for CNVs by RT-qPCR. Primer sequences, position, product size, annealing temperature and TaqMan probes are shown. The CNV status in the SNP-array and in the qPCR and the CNV validation by qPCR are given. **Table S4.** Results of quantitative PCR. The results of the four CNVs per each horse and the outcome of the validation through qPCR are presented.

## Data Availability

The CNVRs identified in this study are available in Additional file 1. The datasets of this study are owned by different research groups or the partners of the EU HORSEGENE consortium (www.horsegene.eu or https://www.biw.kuleuven.be/GENLOG/horsegene/index.html). Data of the breeds Ardenner, Belgian draught horse, German draught, Vlaams paard and Belgian Warmblood are available upon reasonable request from the EU HORSEGENE project consortium, coordinator prof. Nadine Buys (nadine.buys@kuleuven.be), KU Leuven, Belgium. Data belonging to Exmoor ponies is available from the principal investigator Gabriella Lindgren (gabriella.lindgren@slu.se) or publicly available via Figshare (10.6084/m9.figshare.3145759). The Swedish Warmblood dataset was generated and analysed in collaboration with the Swedish Warmblood Association and has a commercial value for them. The SWB horse data is therefore available from the principal investigator Sofia Mikko (sofia.mikko@slu.se) on reasonable request. Data belonging to Friesian horses is subject to owner consent and is available on reasonable request from the principal investigator Bart Ducro (bart.ducro@wur.nl).

## Abbreviations

CNV: Copy number variation
CNVR: Copy number variation region
ECA: *Equus caballus* autosome
FDR: False discovery rate
GAPDH: Glyceraldehyde-3-phosphate dehydrogenase
MNEC670K: Affymetrix Axiom™ Equine genotyping array
Olfr4F21: Olfactory receptor 4F21-like
OR10G2: Olfactory receptor 10G2
OR8S1: Olfactory receptor family 8 subfamily S member 1
PANTHER: Protein analysis through evolutionary relationships
PCA: Principal component analysis
RT-qPCR: Real-time quantitative polymerase chain reaction
SNP: Single nucleotide polymorphism
SV2C: Synaptic vesicle glycoprotein 2C
VEP: Variant effect predictor
